# Hybrid annuloplasty ring procedure for tricuspid valve replacement of Bjork Fontans

**DOI:** 10.1016/j.ijcchd.2026.100684

**Published:** 2026-05-26

**Authors:** Adelle Levi, Daniel Goshen, Glen Van Arsdell, Morris Salem, Reshma Biniwale, Ming Sing Si, Shyamasundar Balasubramanya, Daniel S. Levi, Jamil Aboulhosn

**Affiliations:** a Ahmanson/UCLA Adult Congenital Heart Center, David Geffen School of Medicine at UCLA, United States; b Congenital Cardiac Surgery Program, David Geffen School of Medicine at UCLA, United States; c Division of Pediatric Cardiology, Kaiser Permanente Southern California, United States; d Division of Pediatric Cardiology, Mattel Childrens Hospital, David Geffen School of Medicine at UCLA, United States

## Abstract

**Background:**

Prosthetic ‘tricuspid valve’ placement in Bjork Fontan patients born with tricuspid atresia can transition these patients to a biventricular circulation.

**Objective:**

Describe intermediate term results of hybrid prosthetic valve placement in Bjork Fontans using annuloplasty ring placement around the Bjork connection followed by transcatheter valve.

**Methods:**

A retrospective review of hybrid attempts to valve large Bjork Fontans at UCLA. Only Bjorks that had failed balloon sizing for transcatheter valve placement or had prohibitively large dimensions on cross sectional imaging were included. All had attempted surgical placement of a non-circumferential annuloplasty ring around the Bjork Fontan and subsequent Sapien valve implant.

**Results:**

Median age of the five patients was 40-years. Three had successful placement of annuloplasty rings with a 30 mm (n = 2) or a 34 mm Edwards Physio Annuloplasty ring followed by successful valving with 29 Sapien 3 valves. In two patients, attempts to place a surgical ring resulted in bleeding requiring cardiopulmonary bypass and surgical valve placement. The median follow-up time was 28.7 months. All patients had a significant decrease in CVP and were alive with improvement in their NYHA class to I at most recent follow-up. There was no evidence of valve dysfunction seen on follow up ECHO or cross-sectional imaging.

**Conclusion:**

Bjork Fontan patients often have connections too large for available balloon expandable valves. These patients can be treated with a hybrid incomplete annuloplasty ring placement to enable transcatheter valve placement.

## Highlights

• Transcatheter hybrid Björk valve placement is feasible and beneficial.
• Central venous pressure decreases immediately after the valve placement.
• Intermediate follow-up shows sustained benefit of Björk valve placement.

## Graphical abstract

**Figure:**
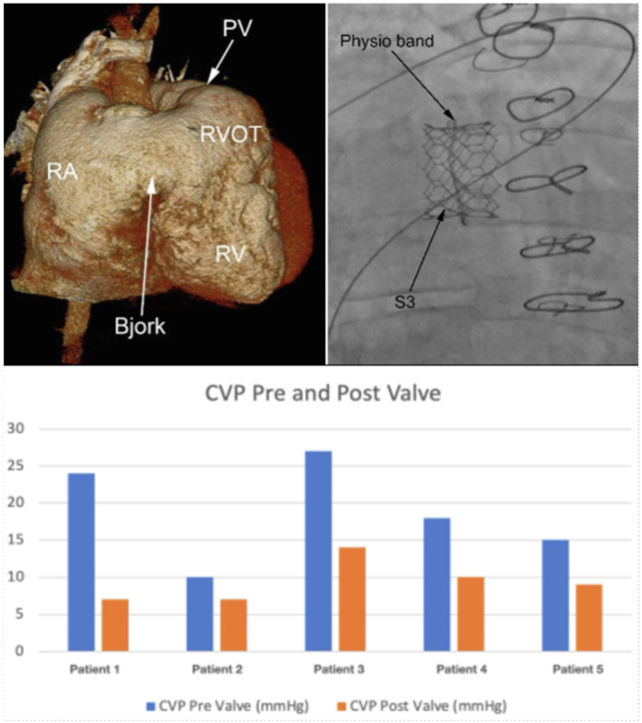

## 1 Background

**Fig. 1:**
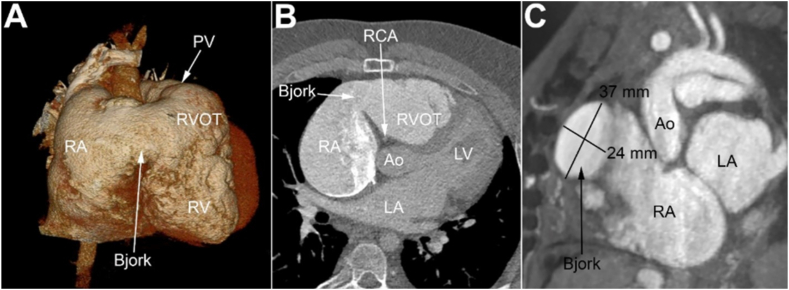
A) ECG gated cardiac CT angiogram with 3-D reconstruction, coronal view, demonstrating a severely dilated right atrium (RA), the Bjork connection between the RA and right ventricular outflow tract (RVOT). Also labeled are the right ventricle (RV) and the location of the pulmonary valve (PV). B) Axial view of the Bjork connection and its relationship to the right coronary artery (RCA), aortic root (Ao). Also labeled are the left atrium (LA) and left ventricle (LV). C) Sagittal view of a cardiac MRI with Feraheme demonstrating the elliptical shape of the large Bjork connection.

The Bjork Fontan modification was originally performed by Dr Viking Bjork and colleagues in 1979 on three children with tricuspid atresia [1]. The original operation consisted of surgical anastomosis of the right atrial appendage (RAA) to the right ventricular outflow tract (RVOT) with the aid of a pericardial patch (Fig. 1). The right ventricle, if not severely hypoplastic, is utilized as a pumping chamber. In the original description by Bjork, the RAA to RVOT connection was non-valved; however, modifications thereafter included placement of a valved conduit from the right atrium (RA) to the RVOT. By the late 1980's hemodynamic and survival outcomes data demonstrated no appreciable advantage to the Bjork modification over the more traditional right atrial to pulmonary artery Fontan, with RA to RVOT conduit stenosis being a long-term complication requiring reoperation [2]. Moreover, the non-valved RAA to RVOT types resulted in hemodynamics mimicking severe right sided atrioventricular valve regurgitation without improvement in cardiac output or central venous pressure when compared to the traditional Fontan operation [3,4]. Hence, the Bjork modification of the Fontan operation was largely abandoned in favor of lateral tunnel and extra-cardiac Fontan modifications. With the advent of transcatheter valve replacement, the valved conduit types of the Bjork could be treated with balloon expandable transcatheter heart valves to relieve stenosis and regurgitation [5]. The development of self-expanding stent platforms has also allowed for valve placement in large non-valved RAA to RVOT modifications [6].

**Fig. 2:**
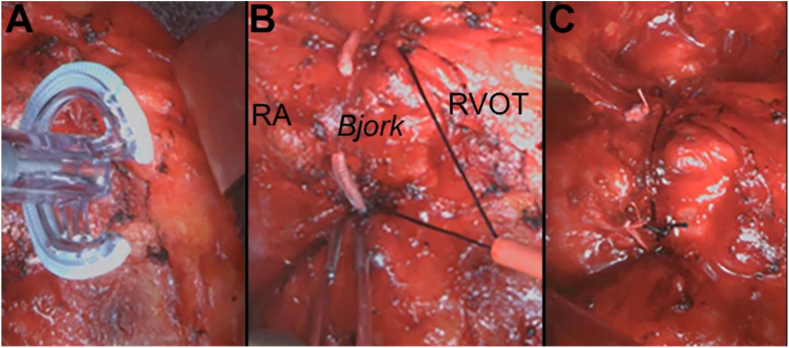
A) Intraoperative picture of tricuspid ring to be placed around the Bjork connection. B) Intraoperative picture of the tricuspid ring placed around the Bjork connection, the right atrium (RA) and right ventricular outflow tract (RVOT) are labeled. C) Intraoperative picture of Bjork after transcatheter deployment of Sapien 3 within the Bjork connection stabilized externally by the tricuspid ring.

This case series details our single center experience with a hybrid approach to allow for transcatheter valve replacement for even the most dilated Fontan connections. This has allowed for placement of competent right atrioventricular valve within the most severely dilated and regurgitant, but unobstructed, RAA to RVOT connections. All of these patients were adults who had Bjork Fontans as children but had developed signs and symptoms of right sided congestive heart failure and volume overload as adults (Fig. 2).

## 2 Methods

**Fig. 3:**
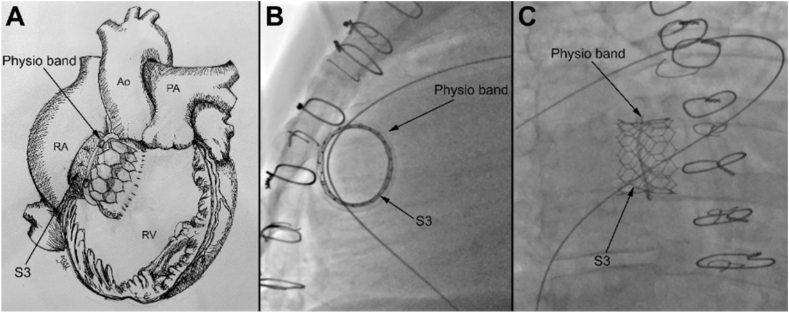
A) artistic rendering of the hybrid operation with the Physio-band placed around the Bjork connection to create a landing zone for the transcatheter heart valve (THV). B) Fluoroscopy in the lateral projection showing the position of the Sapien 3 (S3) within the rigid annuloplasty band (Physio band), C) Antero-posterior view.

**Fig. 4:**
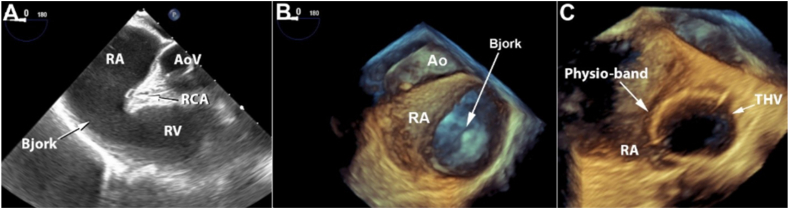
A) Intra-operative transesophageal echocardiography demonstrating the non-valve Bjork connection from the RA to the RV, the right coronary artery (RCA) is labeled as is the aortic valve (AoV). B) 3-D reconstruction of multiplanar TEE imaging showing the elliptical shape of the large Bjork connection as seen from the RA view. C) TEE 3-D reconstruction following hybrid Physio-band and THV placement as seen from the RA view.

A retrospective review of all hybrid attempts to valve RAA to RVOT Bjork Fontans at the University of California, Los Angeles (UCLA) was performed. Only Bjork Fontans that were not considered to have landing zones for balloon expandable transcatheter valves were included; all these patients failed balloon sizing or had prohibitively large dimensions on cross sectional imaging. The five patients included in this series all had attempted surgical placement of a non-circumferential annuloplasty ring (Edwards Physio band, Edwards Life-Sciences, Irvine CA) around the Bjork Fontan and subsequent transcatheter valve in ring placement using the Sapien 3 (Edwards Life-Sciences, Irvine CA) as part of the same procedure (Fig. 2, Fig. 3, Fig. 4). For each patient, basic demographics as well as pre- and post-operative ECHO, cardiac catheterization and exercise testing was collected, and operative and intermediate-term outcomes were described. The study was performed with local IRB approval.

## 3 Results

The median age of the five patients was 40 years (range, 32-43 years) (Table 1). All of the patients had Bjork Fontans placed between the ages of 10-months to 7-years. Three of the five patients had Bjork Fontans placed at 3-4 years of age. There was a wide range of right ventricular end diastolic indexed volumes (RVEDVi). By MRI, RVEDVi ranged from 44 ml to 235 ml/m2. The patient with RVEDVi of 235 ml/m2 had an RVEF of less than 40% and only one patient had greater than mild pulmonary valve regurgitation. This patient had a pulmonary valve regurgitant fraction of 61% and an RVEDVi of 149 ml/m2, and ultimately received a transcatheter pulmonary valve at the time of hybrid transcatheter Bjork TVR (both valves replaced using the hybrid technique described in this manuscript).

**Table 1:** Baseline patient characteristics.

Characteristic	Patient 1	Patient 2	Patient 3	Patient 4	Patient 5
**Age (yrs)**	46	42	34	44	40
**Sex**	Male	Female	Female	Female	Female
**Weight (lbs)**	229.2	113	168	180	142
**NYHA Class**	IV	III	II	II	IV
**Oral diuretic number**	2	0	0	1	1
**Age at Fontan (yrs)**	7	4	3	3	10 months
**Age at Hybrid valve (yrs)**	43	40	32	41	40
**ECHO**					
**LVEF**	55-60%	55-60%	57%	60%	55%
**RA size**	Severely enlarged	Severely enlarged	Severely enlarged	Severely enlarged	Severely enlarged
**RV Size**	Moderately enlarged	Moderately enlarged	Normal	Midly enlarged	Normal
**MRI**					
**RVEDi (mL/m^2)**	235	148.6	64	133	44
**RVEF**	24%	43.50%	67%	43%	57%
**Pulmonary valve Regurgitation**	3%	61%	1%	1.0%	0%
**CATH**					
**SVC (mmHg)**	24	10	27	18	15
**RV (mmHg)**	27/20	17/10	24/20	28/6/15	15/9
**LV (mmHg)**	80/10	98/6	82/12		122/7
**PA (mmHg)**	24/12	17/13	17/13/15	26/14/19	15/10/13
**Cardiac Output (L/min)**	5.8	3.4	2.3	2.6	3.8
**PVR (Wood units)**	1.1	1/1	1.8	3.1	1.9

RVEDVi- Right ventricular end diastolic volume indexed, RVEF- Right ventricular ejection fraction, SVC- superior vena cava, RV- right ventricle, LV- left ventricle, PA-pulmonary artery, PVR-pulmonary vascular resistance.

Three of the five patients had successful placement of non-circumferential annuloplasty rings around the Bjork Fontan connection. Patients received a 30 mm (n = 2) or a 34 mm (n = 1) Edwards Physio Annuloplasty ring followed by successful transfemoral placement of a 29 mm Sapien 3 valve within the ring (Table 2). As above, one patient with a dilated right ventricle and significant pulmonary valve regurgitation received a simultaneous Sapien pulmonary valve replacement as well.

**Table 2:** Patient outcomes.

Operative/Perioperative data	Patient 1	Patient 2	Patient 3	Patient 4	Patient 5
**Type of Bjork Valve**	29 Sapien 3	29 Sapien 3	33 Mitris	29 Mitris	29 Sapien 3
**Type of Bjork Ring**	34 mm CE Physio band	30 mm CE Physio band	None	None	30 mm CE Physio band
**Complications**	None	Stent Strut caused bleeding to MPA requiring surgical repair on bypass	Bleeding on chest entry requiring CPB and surgical valve placement	Bleeding on chest entry requiring CPB and surgical valve placement	Post-op afib treated with amiodarone
**Hospital Stay (days)**	7	5	12	9	7
**ICU Stay (days)**	3	2	5	6	6
**Chest Tube (days)**	3	4	2	6	6
**Outcome post discharge**					
**Follow-up duration (months)**	42	35	24	29	2
**NYHA Class**	I	I	I	I	I
**Oral diuretic quantity**	2	0	0	1	1
**ECHO**					
**LVEF**	50-55%	60-65%	60.0%	58%	57%
**RA size**	Severely enlarged	Severely enlarged	Severely enlarged	Severely enlarged	Moderate enlarged
**RV Size**	Moderately enlarged	Normal	Normal	Normal	Midly enlarged

In two patients, attempts to place a surgical ring around the Bjork Fontan resulted in surgical bleeding requiring institution of cardiopulmonary bypass. Although both of these patients were early in our series, dissection of the Bjork Fontan to the point at which a physio ring could be placed required meticulous attention to tissue planes during dissection. These two patients had institution of cardiopulmonary bypass and received surgical 29 and 33 mm Mitris valves. The patient that also received a pulmonary valve had pulmonary artery injury that was repaired surgically.

Transesophageal echocardiography (TEE) and fluoroscopy were used to guide all procedures. After tricuspid valve replacement, TEE demonstrated competent and unobstructed Sapien 3 prostheses without significant change to qualitative right ventricular systolic function or left ventricular size or systolic function. Comprehensive TEE assessment of other valves did not demonstrate any appreciable change post Sapien 3 valve implantation.

**Fig. 5:**
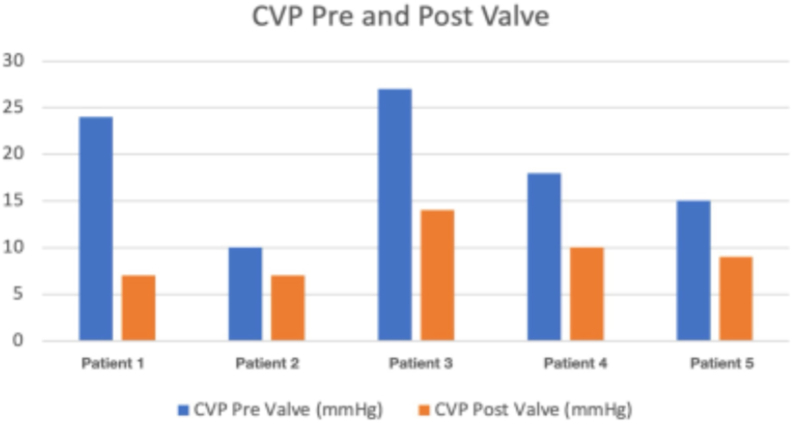
Central venous pressure (CVP) during the hybrid procedure, pre valve implantation (blue bars) and shortly after valve implantation (orange bars). All patients had a reduction in CVP.

There were no operative mortalities. All patients had a decrease in their central venous pressure (CVP) immediately after valve implantation from a mean of 19 mmHg to 9 mmHg (Fig. 5). The median hospital stay was 7 days (range 5-12 days) without need for re-operation or further intervention (Table 2). One patient had post-operative atrial fibrillation that required cardioversion.

The median follow-up time was 28.7 months (range, 0.5- 38.2 months). All patients were alive with improvement in their NYHA class to I at most recent follow-up (original NYHA classifications were 2-4). There was no evidence of valve dysfunction seen on follow up transthoracic echocardiogram or cross-sectional imaging, although imaging of the valved Bjork connection by transthoracic echo is challenging given the retrosternal location of the valved complex.

The first patient in this series reported increased need for an oral loop diuretic at his follow-up visit 34 months post implantation. Transthoracic echocardiographic imaging did not visualize the prosthetic valve well. Thus, a catheterization was performed and intracardiac echo imaging demonstrated moderate prosthetic valve regurgitation, therefore a 29 S^3^ valve in valve implant was performed without complications.

## 4 Discussion

The Fontan palliation has allowed infants born with single ventricle physiology to survive into adulthood but it does come with numerous long-term sequelae and complications, mostly related to a chronic low cardiac output state and chronically elevated central venous pressure that are directly related to the presence of only one functional ventricle. The Bjork modification which was developed for use in patients with tricuspid atresia includes the right ventricle within the Fontan circulation in an attempt to use this second pump to improve cardiac output. However, the Bjork modification was largely abandoned by the early 1990's due to the resultant severe right atrioventricular valve regurgitation in non-valved RAA to RVOT modifications. The Bjork modifications that utilized valved RA to RVOT conduits were also subject to progressive stenosis and regurgitation requiring reoperation. Hence, it was largely determined that the Bjork modification did not provide significant advantages over other Fontan types that completely excluded the right ventricle, neither in improving cardiac output or central venous pressure. Long-term follow-up of children that underwent Bjork Fontan modifications have shown expected complications as seen in other Fontan subtypes, those being related to chronically elevated central venous pressure with liver and other organ congestion, decreased cardiac output, and atrial arrhythmias [7].

This case series describes a novel hybrid procedure for placing a competent transcatheter heart valve within dilated RAA to RVOT Bjork connections in five adult patients with signs and symptoms of volume overload and right heart failure. Three of the five operations did not require cardiopulmonary bypass. The two that did require cardiopulmonary bypass did so due to surgical bleeding during chest entry given the proximity of the RAA/RVOT to the sternum. Additionally, one patient required repair of a pulmonary artery injury related to transcatheter valve placement. Nonetheless, there were no operative mortalities and the central venous pressure decreased in all patients. Moreover, TEE imaging demonstrated that all valves were competent and unobstructed. There was no decrement in ventricular systolic function with valve placement and all patients did well post-operatively and have had an improvement in functional capacity, none have required hospitalization for decompensated heart failure since the operation. One patient underwent elective valve in valve replacement more than three years post initial implantation, this was an uncomplicated procedure and a 29 S^3^ was easily implanted with full volume within the existing complex.

Given the positive results of “valving the Bjork” described here and by other investigators, it is reasonable for the congenital cardiac community to reconsider the abandoned original RAA to RVOT non-valved Bjork modification as a means of establishing a long-term biventricular circulation in ‘single ventricle’ physiology. Viking Bjork and colleagues stated in the original description that a patient with a severely hypoplastic right ventricle may not be a candidate for this operation and that likely still holds true. However, we noted that in all of these adult patients that had been subjected to chronic right ventricular volume overload by the severely regurgitant non-valved RAA to RVOT communication, the right ventricles were of adequate size and function to handle all forward cardiac output. Could the non-valved Bjork modification be utilized in patients with tricuspid atresia and hypoplastic right ventricles to chronically volume overload the right ventricle with resultant dilation to an adequate size to allow for pure transcatheter or hybrid transcatheter valving techniques, with the end result being a functional biventricular circulation? The experience with the five cases described here certainly suggests that this approach may be feasible and favorable given the long-term sequelae and complications of the Fontan operation in single ventricle physiology. It is certainly worthy of further consideration and debate in the era of transcatheter valve replacement and hybrid operations.

## 5 Limitations

This is a small case series that included only 5 patients with this rare condition. The operative data from childhood was not available for the patients. The imaging data from childhood was also not available, therefore, we could not ascertain right ventricular size or function during infancy or childhood.

## 6 Conclusions

Bjork Fontan patients often have dilated RAA to RVOT connections not amenable to commercially available transcatheter balloon expandable valves. These patients are amenable to hybrid incomplete annuloplasty ring placement around the RAA followed by transcatheter valve in ring placement. There was an immediate reduction in CVP after valve placement by a mean of 10 mmHg on intra-procedural hemodynamic monitoring. On intermediate term follow up, all patients had improved NYHA classification and were alive. These findings showing favorable results when converting Bjork type Fontans to a biventricular circulation with a competent ‘tricuspid valve’ beg the question of whether the Bjork Fontan modification should again be considered for select patients with tricuspid atresia.

## CRediT authorship contribution statement

**Adelle Levi:** Conceptualization, Data curation, Formal analysis, Investigation, Methodology, Project administration, Writing – original draft. **Daniel Goshen:** Writing – review & editing. **Glen Van Arsdell:** Writing – review & editing. **Morris Salem:** Writing – review & editing. **Reshma Biniwale:** Writing – review & editing. **Ming Sing Si:** Writing – review & editing. **Shyamasundar Balasubramanya:** Writing – review & editing. **Daniel S. Levi:** Writing – review & editing. **Jamil Aboulhosn:** Conceptualization, Data curation, Formal analysis, Investigation, Methodology, Project administration, Writing – original draft.

## Declaration of competing interest

The authors declare the following financial interests/personal relationships which may be considered as potential competing interests: Jamil Aboulhosn reports a relationship with Edwards Lifesciences Corporation that includes: consulting or advisory. Daniel S Levi reports a relationship with Edwards Lifesciences Corporation that includes:. Jamil Aboulhosn reports a relationship with Medtronic Inc that includes: consulting or advisory. Daniel S Levi reports a relationship with Medtronic Inc that includes: consulting or advisory. If there are other authors, they declare that they have no known competing financial interests or personal relationships that could have appeared to influence the work reported in this paper.
